# Two novel mutations in exon 3 of *PHOX2B* gene: think about congenital central hypoventilation syndrome in patients with Hirschsprung disease

**DOI:** 10.1186/s13052-019-0636-8

**Published:** 2019-04-18

**Authors:** Maria Giovanna Paglietti, Claudio Cherchi, Federica Porcaro, Emanuele Agolini, Alessandra Schiavino, Francesca Petreschi, Antonio Novelli, Renato Cutrera

**Affiliations:** 1 Respiratory Unit, Academic Department of Pediatrics, Bambino Gesù Children’s Hospital, IRCCS, Piazza di Sant’Onofrio 4, 00165 Rome, Italy; 2 Laboratory of Medical Genetics, Bambino Gesù Children’s Hospital, IRCCS, Rome, Italy

**Keywords:** Congenital central hypoventilation syndrome, *PHOX2B* gene, NPARMs, C.255_256delCT, C.780dupT, Genotype-fenotype correlation

## Abstract

**Background:**

Congenital central hypoventilation syndrome (CCHS) is characterized by alveolar hypoventilation increasing during sleep and affected patients are unable to perceive and respond to hypercarbia with increased ventilation and arousal during sleep. *PHOX2B* gene mutations are considered as responsible for CCHS. Most of patients with CCHS are heterozygous for polyalanine expansion mutations (PARMs) in exon 3, but 10% of patients with classic CCHS are heterozygous for non-polyalanine expansion mutations (NPARMs) of the *PHOX2B* gene.

**Methods:**

Data are collected on 3 patients affected by CCHS who referred to the Paediatric Pulmonology Unit of Bambino Gesù Children’s Hospital (Rome, Italy) for a multidisciplinary follow-up program between 2000 and 2017.

**Results:**

We describe three cases of patients affected by CCHS for which two novel mutations on exon 3 of *PHOX2B* gene were detected.

**Conclusions:**

The description of these novel mutations and related clinical phenotypes allows to expand the knowledge into NPARM spectrum. Since the presence of Hirschsprung disease is related to NPARMs and the number of alanine repeats, we suggest performing CCHS genetic investigation and periodical assessment also in patients without a clear history of CCHS but affected by Hirschsprung disease.

**Trial registration:**

Data are retrospectively collected.

## Introduction

Congenital central hypoventilation syndrome (CCHS) is a rare life-threatening disorder characterized by autonomic dysregulation and alveolar hypoventilation often requiring lifelong ventilatory assistance. As a result of hypoventilation, affected subjects become hypoxemic, hypercarbic and unable to perceive and respond to hypercarbia with increased ventilation and arousal during sleep [1]. More severely affected individuals exhibit hypoventilation both awake and asleep [2]. Nevertheless, hypoventilation increases during sleep, particularly in the non-REM phase, in which the autonomic control of breathing predominates [3].

*PHOX2B* gene mutations are considered as responsible for CCHS [2] and over 1000 cases of *PHOX2B* mutation-confirmed CCHS are reported [2]. The normal genotype of *PHOX2B* gene is reported as 20/20 polyalanine repeat numbers. It encodes a transcription factor responsible for the regulation of genes involved in the development of the autonomic nervous system [4]. Over 90% of CCHS cases are heterozygous for polyalanine expansion mutations (PARMs) in exon 3 of *PHOX2B* gene producing genotypes of 20/24 to 20/33 [5]. Most of these mutations are de novo and 20/25, 20/26 and 20/27 genotypes are the most common described [5].

The remaining 10% of patients with classic CCHS are heterozygous for non-polyalanine expansion mutations (NPARMs) founded at the end of exon 2 or in exon 3 of the *PHOX2B* gene. Most of these mutations are de novo and approximately 10–25% of asymptomatic parents are responsible for transmission to their offspring [6].

The purpose of our study is to describe two novel mutations on exon 3 of *PHOX2B* gene expanding the knowledge into NPARM spectrum.

## Methods

Data are retrospectively collected on 3 patients affected by CCHS who referred to the Paediatric Pulmonology Unit of Bambino Gesù Children’s Hospital (Rome, Italy) for a multidisciplinary follow-up program between 2000 and 2017.

Demographic data, medical histories and results of diagnostic testing were obtained from electronic medical record review with approval from the institutional review board.

Nocturnal polysomnography, transcutaneous capnography, echocardiography were performed at baseline in patients with history of apnea, reduction in blood oxygen saturation and cyanosis on falling asleep in order to detect central hypoventilation or heart diseases.

Neurological, ophthalmological, maxillofacial, psychologist and swallowing evaluations were also considered in our analysis.

Blood samples were obtained with informed consent of the probands or their parents to perform genetic analysis when central hypoventilation and hypercarbia were confirmed on nocturnal polysomnography and transcutaneous capnography.

Molecular analysis of *PHOX2B* gene was performed by Sanger sequencing (primer sequence and PCR conditions are available upon request) on a genomic DNA sample extracted from whole blood of patients. The identified mutations were checked in parents for segregation by direct sequencing.

Once the diagnosis was obtained, patients were included in a follow-up program providing ventilatory assessment during awake and asleep states, neurocognitive assessment, electroencephalography, continuous ECG monitoring and echocardiogram every 4 months in infants under the age of two years and every 6 months in those under the age of 3 years; then, all the investigations were annually performed when clinical stability was present [2].

Abdominal ultrasound, urine cathecholamines and fecal occult blood testing were carried out every 4 months in first 2 years, then every 6 months in infant under the age of 3 years; then investigations were annually performed to identify neuroblastomas [2].

## Results

### Case 1

A male caucasian child, born at 37 weeks of gestational age with birth weight 2980 g.

He had family history of Hirschsprung disease (HSCR) occurring in his mother. His past medical history was positive for delayed meconium and distended abdomen in the second day of life. A barium contrast enema study has been performed and the result suggested the hypothesis of HSCR then confirmed by rectal suction biopsy and acetylcholinesterase immune-histochemical examination. The infant underwent to surgical repair through a laparoscopic pull through procedure. During hospitalization in Surgical Department, the patient had recurrent apneas and cyanosis during sleep requiring investigations as brain and heart ultrasound and chest X-ray that were all normal. Due to family and personal history of HSCR and reported respiratory complaints, the child referred to our Pulmonology Unit when he was 2-months in order to exclude the diagnosis of CCHS. Nocturnal polysomnography detected central hypoventilation and transcutaneous capnography revealed increased levels of CO2 (mean value 53.2 mmHg). Sequencing analysis revealed a normal number of alanine repeats (20/20) in *PHOX2B* gene but allowed to identify a novel likely deleterious heterozygous variant in exon 3 corresponding to the frameshift mutation c.255_256delCT (p.Phe86HisfsTer91), resulting in a premature stop codon. Genetic analysis, extended to child’s parents, confirmed that his mother carried the same mutation. To exclude the presence of a mosaicism, the variant has been confirmed, on a maternal DNA sample isolated from buccal mucosa, in heterozygous state with a ratio between mutant and normal allele comparable to that observed on the blood DNA. During hospitalization, the child started Non-Invasive Ventilation (NIV) with consequent clinical and gas exchange improvement.

Currently, no other clinical problems are emerging during periodical follow-up program.

### Case 2

A 34-year-old caucasian female with past medical history of surgically treated HSCR.

No other significant symptoms were reported. As her son received the diagnosis of CCHS (see the case described above), she underwent to nocturnal polysomnography that was normal. Nevertheless, the sequencing analysis of the *PHOX2B* gene was carried out and the same mutation (c.255_256delCT) was singularly detected. Even though the same mutation was detected, the phenotype observed in her son was more severe with early onset of symptoms.

### Case 3

A female caucasian child, born at 36 weeks of gestational age with birth weight 2760 g.

Her mother had history of several miscarriage. She had neonatal hypoxia and respiratory failure at birth needing hypothermia treatment and invasive mechanical ventilation (IMV) in the first days of life. Because weaning from mechanical ventilation failed, tracheostomy has been positioned in Neonatal Intensive Care Unit (NICU). Because of recurrent apneas and cyanosis during sleep despite tracheostomy, polysomnography was performed and showed central hypoventilation. In addition, she had delayed meconium suggestive of HSCR, subsequently confirmed by rectal suction biopsy and acetylcholinesterase immune-histochemical examination.

The patient showed recurrent seizures with negative neurological examination (sensory neuron and motor response evaluation). Electroencephalography (EEG) was normal and brain MRI showed a mild brain injury due to neonatal hypoxia. Therefore, glucose monitoring was considered and very low glucose levels were observed during all critical episodes that were not related to meals. Glycogen storage disease (GSD), GH deficiency and adrenal insufficiency were excluded because the absence of chetonuria. Normal values of urine organic acid, serum amino acid panel, plasma acylcarnitine profile, insulin and C-peptide, allowed to exclude respectively beta oxidation disorders and hyperinsulinism.

Because of dysphagia, gastrostomy was required and continuous enteral nutrition was started, even though recurrent hypoglycemic events continued to be present.

Moreover, the baby presented several episodes of breathing hold spell triggered by pain, frustration and fear occurring also during mechanical ventilation.

Due to the described clinical picture, genetic examination was performed to detect CCHS when she was three months old. Sequencing analysis excluded an alanine expansion (20/20) in *PHOX2B* gene but revealed a novel likely deleterious heterozygous variant in exon 3, c.780dup leading to 44 amino acids elongated protein. The presence of the mutation was excluded in healthy patient’s parents by direct sequencing. During hospitalization the child started weaning from IMV and spontaneous awake breathing has been reached.

## Discussion

In this study we describe two novel mutations on exon 3 of *PHOX2B* gene expanding the knowledge into NPARM spectrum: c.255_256delCT and c.780dup.

The first one has been detected in a child and his mother. As most of NPARMs, both patients had history of HSCR. Nevertheless, despite the same detected mutation, the clinical course was different and characterized by delayed meconium and recurrent apneas and cyanosis in the first month of life with abnormal nocturnal monitoring in the affected child. Differently, his mother reported only intestinal involvement and no hypoventilation was actually detected on nocturnal monitoring. This observation suggests that environmental factors can contribute to the variability of clinical expression of the disease. Moreover, as we consider the benign evolution of the ventilation in patient 2 as uncommon feature of described disease, we suggest, in any case, yearly assessment of asymptomatic patients in order to identify the late onset symptoms.

In fact, a genotype-phenotype correlation is well described and symptoms severity – including continuous ventilatory dependence, heart rhythm disorders, association with HSCR and neural crest tumors – is closely related to NPARMs and to the number of alanine repeats among subjects with PARMs [7].

Indeed HSCR – that occurs among 20% of cases of CCHS – is more prevalent among NPARMs subjects (87–100%) than among those with PARMs (13–20%) [8]. Short segment involvement (rectosigmoid) is more common, but long-segment aganglionosis is also described [9].

The incidence of HSCR is approximately one in 5.000 live births in general population [10], while that of CCHS is one in 200.000 live birth [11]. We are consciousness that genetic analysis for CCHS is not feasible in all patients with HSCR, but it should be considered when respiratory and/or dysautonomic symptoms occur. Cases number 1 and 3 confirm the above conclusion, even if our case number 2 would not have been recognized without her son’s diagnosis.

Tumors derived from the neural crest (neuroblastoma, ganglioneuroma, ganglioneuroblastoma) occur more frequently among individuals with NPARMs (50%) than among those with PARMs (1%) [8]. Among PARMs subjects, only patients with the 20/29 and 20/33 genotypes have been identified to have tumors of neural crest origin [2, 7, 8, 12].

Even if classic CCHS presentation is typically in the neonatal period, rarely, a milder later-onset (LO-CCHS) presentation is described in toddlers, children and adults.

LO-CCHS reflects the variable penetrance of *PHOX2B* mutations with the mild genotypes 20/24 and 20/25 or, rarely, NPARM requiring an environmental cofactor to unmask the phenotype [2].

The second described mutation has been firstly reported: even if respiratory failure at birth was probably influenced by neonatal hypoxia, the subsequent recurrent and severe apnea ad cyanosis episodes were attributable to CCHS. The presence of severe hypoglycemia associated to seizures as well as the breathing hold spells occurring also during mechanical ventilation and dysphagia were interpreted as autonomic nervous system impairment yet described in NPARMs carrier patients [11, 13, 14, 15]. As previous clinical cases with NPARMs, also the third patient had HSCR. According to these observations, it is possible that the last described mutation leads to a severe phenotype with more autonomic impairment.

## Conclusion

To the best of our knowledge, literature suggests that CCHS has to be suspected in cases of centrally mediated alveolar hypoventilation and/or cyanosis or seizures arisen after anesthetics or CNS depressants administration, recent severe lung infection or treatment of obstructive sleep apnea [4, 7]. Our study suggests performing CCHS genetic investigation and periodical assessment also in patients without a clear history of CCHS but affected by Hirschsprung disease.

## Abbreviations

CCHS: Central congenital hypoventilation syndrome
EEG: Electroencephalography
HSCR: Hirschsprung disease
IMV: Invasive mechanical ventilation
LO–CCHS: Late-onset central congenital hypoventilation syndrome
NICU: Neonatal Intensive Care Unit
NIV: Non-Invasive Ventilation
NPARMs: Non-polyalanine repeat expansions mutations
PARMs: Polyalanine repeat expansions mutations
